# Transsphenoidal pituitary adenoma resection: do early post-operative cortisol levels predict permanent long-term hypocortisolism?

**DOI:** 10.1007/s10143-021-01643-w

**Published:** 2021-09-20

**Authors:** Vicki M. Butenschoen, Alexander von Werder, Stefanie Bette, Veronika Schmette, Nina Schwendinger, Bernhard Meyer, Jens Gempt

**Affiliations:** 1 Department of Neurosurgery, Klinikum Rechts Der Isar, Technical University Munich, Ismaningerstr. 22, 81675 Munich, Germany; 2 II. Medizinische Klinik Und Poliklinik, Klinikum Rechts Der Isar, Technische Universität München, Munich, Germany; 3 Abteilung Für Diagnostische Und Interventionelle Neuroradiologie, Universitätsklinikum Augsburg, Augsburg, Germany

**Keywords:** Pituitary adenoma, Transsphenoidal surgery, Hypocortisolism

## Abstract

Transsphenoidal surgery provides a minimal invasive treatment for pituitary adenoma. Our aim is to evaluate the endocrinological outcomes after adenoma resection focusing on the corticotroph function, and to identify prognostic factors for an impaired hypothalamic–pituitary–adrenal-axis function (HPA) and the reliability of postoperative early morning serum cortisol measurements. We performed a retrospective analysis of all patients treated for pituitary adenoma from April 2006 to January 2019 in our neurosurgical department. Pituitary function was assessed pre- and postoperatively as well as at 6 weeks to 12 weeks and at 1-year follow-up. Two hundred eleven patients were included. Nine percent of the patients recovered from a preoperative adrenal insufficiency, 10.4% developed a new need for hormone substitution, and a long-term deficiency of the hypothalamic–pituitary–adrenal-axis was observed in 30.9%. Cortisol measurements 5 days after surgery had a lower area under the curve (AUC) than cortisol levels detected after 6 to 12 weeks (AUC 0.740 vs. AUC 0.808) in predicting an intact corticotrope function. The cut-off value determined for cortisol measured after 6 weeks was 6.95 µg/dl (sensitivity of 94%, specificity of 68%). Postoperative early morning cortisol levels seem to be less sensitive and specific in predicting long-term corticotroph function than measurements after 6 weeks and 1 year, emphasizing the importance of endocrine follow-up testing.

## Introduction

Tumors of the pituitary gland account for approximately 15% of all intracranial tumors and the benign pituitary adenoma represent the most common encountered entity (up to 85%) [8, 39, 42, 43] of all sellar lesions. The transnasal transsphenoidal (TSS) operative resection of sellar tumors has firstly been described in 1907 by Schlofer et al. and further developed by Cushing et al. in 1909 [5, 27, 40]. Nowadays, TSS is considered the most appropriate and initial approach for treatment of tumors of the pituitary gland [40, 41]. It can reduce hospital costs by shortening the length of hospital stay (LOH) and presents a safe and effective surgical resection option with fewer complications and better clinical out-comes than the transcranial route [44, 45].

Pituitary adenoma patients may present with mass effect and lead to a compression of the optic chiasm with visual field defects and impaired visual acuity. As the pituitary gland plays a pivotal role in hormone regulation, adenomas can lead to an impaired adrenal pituitary function or hormone hypersecretion such as Acromegaly or M. Cushing due to a hypersecretion of growth hormone (GH) or adrenocorticotropic hormone (ACTH).

Most important is the preservation of an intact hypothalamic–pituitary–adrenal-axis (HPA) regulating in a circadian rhythm and depending on physical stress levels cortisol release through the stimulation of ACTH [15]. Therefore, patients need to be monitored closely for symptoms of hypocortisolism, the most dangerous and life-threatening complication of pituitary deficiency [28], presenting with impaired metabolism such as anorexia, fatigue, hypotension and nausea, and potentially leading to the lethal adrenal crisis including shock and volume depletion called Addison’s crisis [18, 33]. Many studies have focused on the optimal serum cortisol threshold (or cut-off value) to determine whether a patient suffers from hypocortisolism or not, and to prevent patients from adrenal corticotropic deficiency by substituting hydrocortisone [3, 17, 29, 31, 43]. Oral hydrocortisone, the generic pharmaceutical name of cortisol, has a plasma half-life of 1.5 h, a biologic half-life of 8–12 h, and a glucocorticoid potency of 0.8 [13, 36]. Minimum serum values range from 4 to 20 μg/dl (morning serum level, 1 µg/dl = 27,59 nmol/l), but only few conducted studies have been performed and published with a long enough follow-up to establish cortisol supplementation criteria [24, 28, 43].

The optimal cut-off value of morning fasting cortisol level plays a pivotal role in determining the risk for hypocortisolism and needs to be detected early in order to start the optimal treatment, but also to avoid hydrocortisone over-replacement to minimize possible side effects like osteoporosis and impaired glucose tolerance [2, 37]. Regular follow-up testing of the fasting morning cortisol levels is therefore performed before and after surgery, as well as at neurooncological follow-up appointments.

In our study, we focus on the diagnostic value of direct postoperative and follow-up cortisol measurements and possible influencing prognostic factors to detect pituitary hypocortisolism due to surgery-related injury of the pituitary gland, in order to provide the most reliable timing and cortisol cut-off value to predict long-term need of hydrocortisone replacement therapy.

## Methods

We retrospectively analyzed all patients treated via TSS for histologically proven pituitary adenomas from April 2006 to January 2019 in our neurosurgical department in a tertiary referral hospital by two senior attending neurosurgeons.

Inclusion criteria were diagnosed hormone-inactive adenoma of the sellar region, complete data available including complete clinical and endocrinological data such as cortisol serum levels, age > 18 years, minimum endocrinological and clinical follow-up of 1 year after surgery, and surgery performed via a transsphenoidal approach. Patients with other tumors than adenoma of the sellar region and prior intake of prednisolone were excluded from our study. Patients presenting with pituitary apoplexy were included if complete endocrinological testing was performed before surgery and adenoma tissue was identified in the specimens sent to histopathology.

We retrieved demographic factors from our archives such as gender, age, and Karnosfky performance scale (KPS), as well as comorbidities from patient files, pre- and postoperative imaging (magnetic resonance imaging MRI, computed tomography CT) for extent of resection (EOR, gross total resection GTR vs. partial resection (PR)), and perioperative hormone levels of basal cortisol (µg/dl) gained from the patient in the morning (fasting serum values, cobas 8000 analyzer Roche). Urinary free cortisol levels were not included for analysis. Information on the operation performed included microscopic vs. endoscopic approach, length of operation (mean time in minutes), and intra-operative complications (cerebrospinal fluid CSF leak, major bleeding). The minimum clinical and endocrinological follow-up time was 1 year, and patients were monitored for anterior and posterior pituitary deficiency and need for hormone substitution after TSS. Corticotropic deficiency was also diagnosed when the patient presented with clinical symptoms of hypocortisolism after withdrawing or reducing the oral hydrocortisone substitution, even if the basal morning serum cortisol level was above a defined threshold.

Statistical correlation analyzes were performed using the IBM SPSS software (Version 26.0.0.0). Multivariable analyses were conducted using ANOVA and Chi^2^ testing. Cut-off values were determined using the receiver-operator curve (ROC), area under the curve (AUC), and the Youden-Index [46]. Correlation analysis was performed using the Spearman coefficient, and a *p*-value < 0.05 was considered significant.

The presented study meets the ethical standards outlined in the Declaration of Helsinki, ethics approval was obtained before performing the analysis (Ethikkommission der Technischen Universität München, Prof. Dr. Schmidt), and the positive vote was registered under the number 231/20-S. Due to the retrospective nature of the study, informed consent was waived in accordance with the Ethics Commission. All methods were carried out in accordance with relevant guidelines and regulations.

## Results

### Patient population

**Fig. 1:**
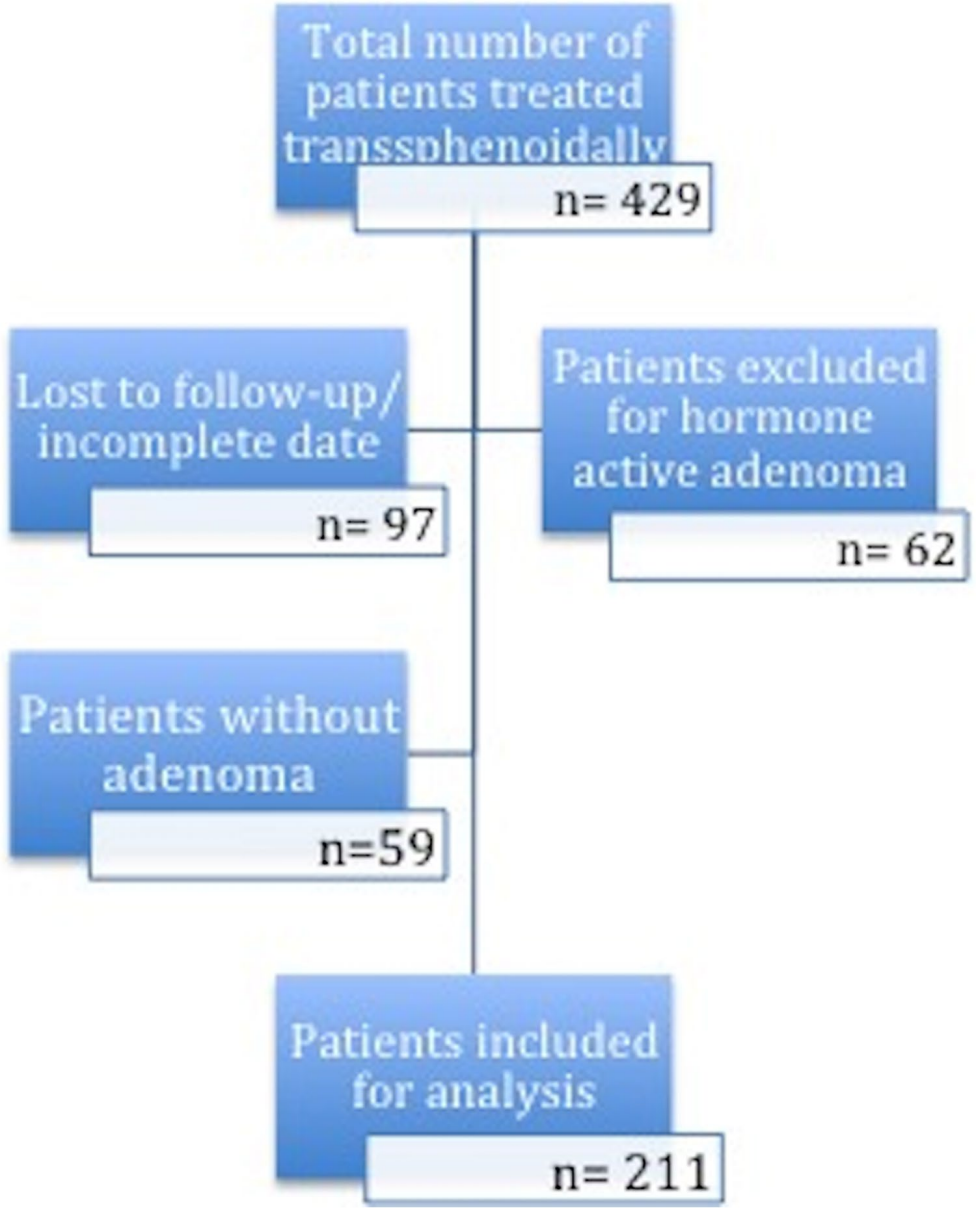
Flowchart describing the number of patients meeting the inclusion and exclusion criteria and showing the number of patients included for analysis (*n* = 211)

We were able to identify 429 patients who were treated via the transsphenoidal approach for tumors of the sellar and parasellar region from April 2006 to January 2019 in our clinical neurosurgical department. Sixty-two patients with hormone-producing adenoma causing Acromegaly and M. Cushing were excluded due to their distinct preoperative cortisol levels (Fig. 1). Fifty-nine patients were excluded due to histopathological diagnosis of tumors other than pituitary adenoma (meningioma, Rathke’s cleft cysts, hypophysitis) due to their distinct clinical outcome. In total, 97 patients were lost to follow-up endocrinological data at 1 year.

We were able to include 211 patients for further analysis. Comparing the numbers over the years, we observed a trend towards increasing numbers of surgeries performed for transnasal pituitary adenoma resection (number of cases included between 2006 and 2012, *n* = 72; 2013–2019, 139 cases).

Median age was 56 years with a range of 20–86 years. Sixty percent (*n* = 127) were male and 40% (*n* = 84) female patients. Median preoperative KPS was 90% (range 20–100%). 97.6% of the patients suffered from pituitary macroadenoma, and only 5 patients presented with progressive hormone-inactive pituitary microadenoma (only selected cases such as patients presenting with tumor growth on follow-up imaging leading to the indication of surgical removal (2.4%)).

### Hormone levels

All patients were given intra- and postoperative hydrocortisone substitution according to the following standard scheme: patients obtained 50 mg of intravenous hydrocortisone injection on induction, followed by immediate initiation of a continuous infusion of hydrocortisone 100 mg/24 h. On postoperative day 1, patients obtained 80 mg (infusion for 24 h). On day 2 after surgery, we continued with an oral administration of 60 mg hydrocortisone (20 mg in the morning, noon, and evening) with continuous reduction of 10 mg per day until the maintenance dosage of 20 mg per day (10 mg in the morning and at noon).

Table 1 describes the preoperative values, postoperative values, and long-term cortisol results after one year of patients grouped by hypocortisolism. Mean preoperative early morning cortisol level was 11.7 µg/dl (range 0.2 to 37.1 μg/dl) and mean postoperative (5 days after TSS) level was 12.6 µg/dl (range 0.2 to 45.1 μg/dl). At 6 weeks to 12 weeks, mean early morning cortisol level was 11.9 µg/dl (range 0.1 to 59.8 µg/dl) and long-term data (minimum 1-year follow-up) revealed a mean basal cortisol level of 11.2 µg/dl (range 0.1–35.1 µg/dl).

**Table 1:** Preoperative hormone levels classified by timing of the analysis and classified in patients with corticotroph deficiency and intact corticotroph function 5 days after surgery (“early postoperative”), 6 to 12 weeks after surgery and 1 year after surgery with 95% CI (confidence interval)

Mean hormone level	Preoperative	Early postoperative	After 6–12 weeks	1 year after surgery
Basal cortisol (μg/dl)@(95% CI)	11.7 (10.7–12.6)	12.6 (11.5–13.8)	11.9 (10.7–13.1)	11.2 (10.2–11.2)
In patients with hypocortisolism	3.99 (3.2–4.8)	8.6 (6.5–10.7)	7.7 (5.4–9.8)	6.01 (4.2–7.8)
In patients without hypocortisolism	15.1 (14.2–16.0)	15.1 (13.7–16.5)	13.4 (12.4–14.5)	13.7 (12.7–14.6)

**Fig. 2:**
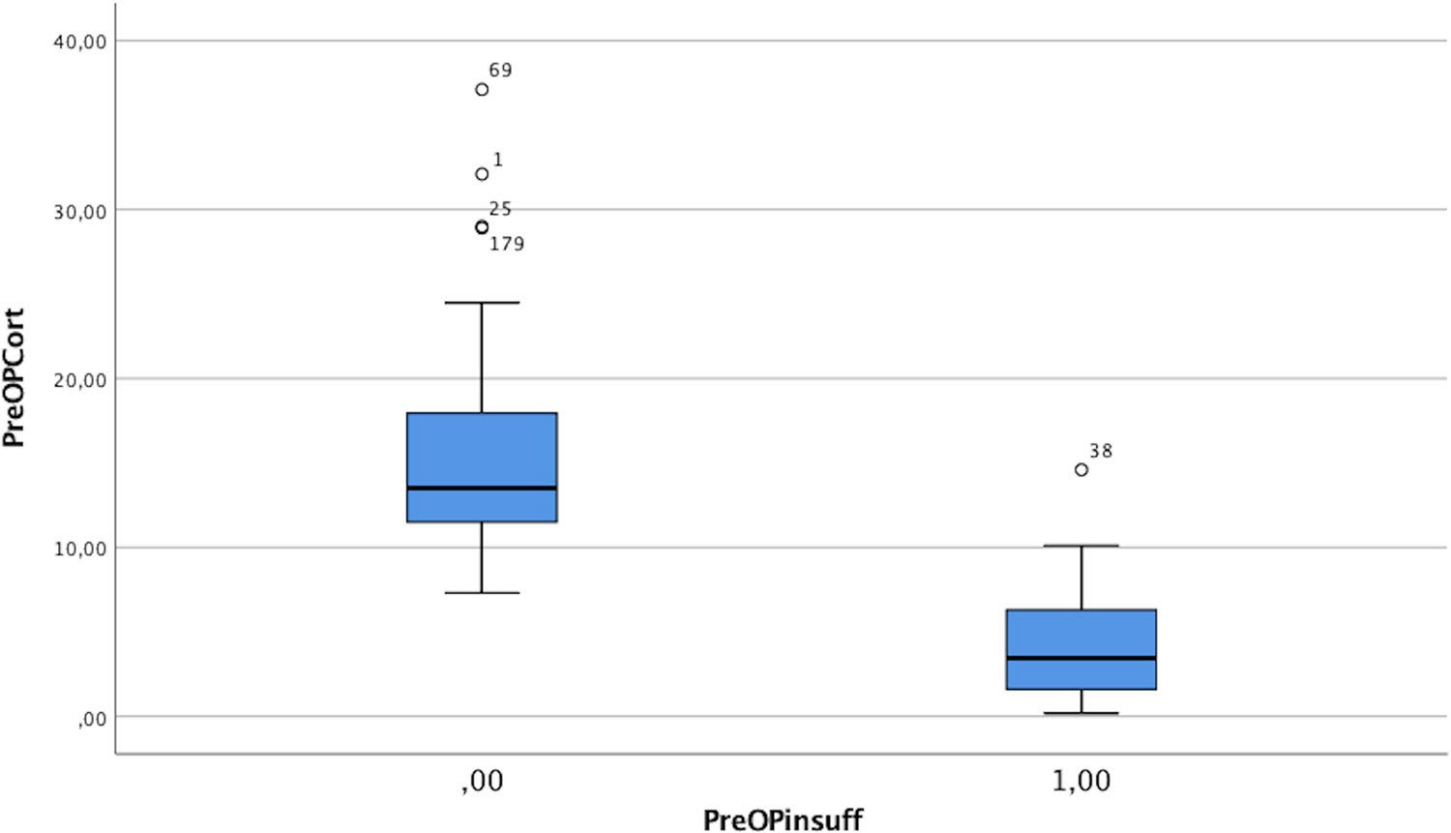
Distribution of basal cortisol levels depending on necessity of hydrocortisone substitution (0: no substitution, 1: hydrocortisone substitution) before surgery (in µg/dl)

**Fig. 3:**
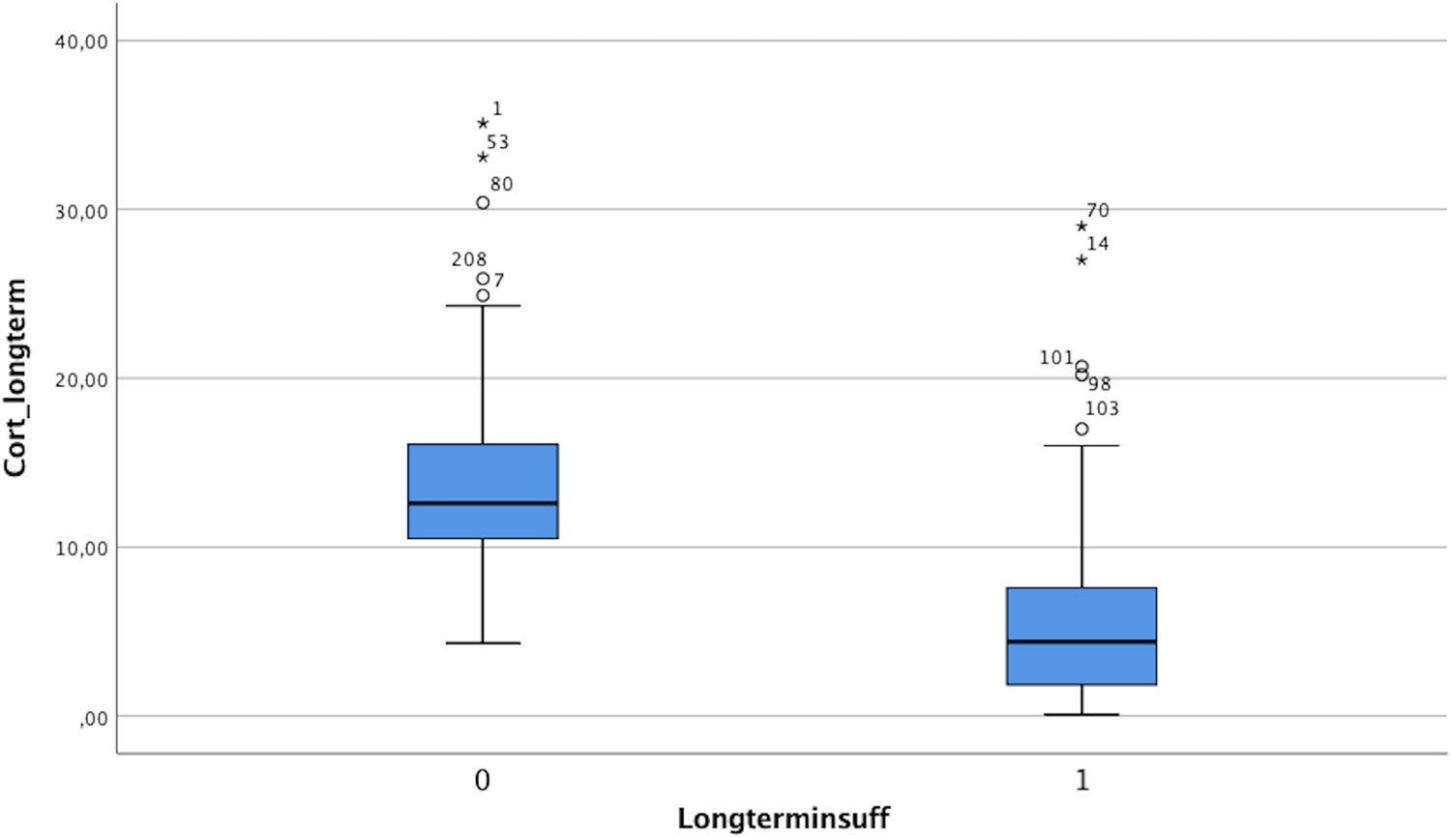
Distribution of basal cortisol levels depending on necessity of hydrocortisone substitution (0: no substitution, 1: hydrocortisone substitution) 1 year after surgery (in µg/dl)

If grouped by clinical hypocortisolism symptoms and need for hydrocortisone substitution, mean basal serum cortisol values in patients with hypocortisolism were 3.99 µg/dl and 15.1 µg/dl. Mean hormone levels of basal cortisol at long-term follow-up (minimum 1 year) were 6.01 µg/dl for patients with long-term need of hydrocortisone substitution and 13.7 µg/dl for the healthy counterpart without replacement therapy (Figs. 2 and 3, Table 1).

### Clinical and surgical outcome

The overall clinical outcome showed satisfying postoperative results. Median postoperative KPS of our patients was 90% (range 40–100%). Mean duration of surgery was 84 min, ranging from 24 to 489 min. Of all performed surgeries, 68.1% of the TSS were performed with a microscope, and in 31.9% of the cases with a 0° or 30° endoscope. We did not identify any significant differences in terms of complications or clinical outcome between patients operated with the endoscope or microscopical approach.

Overall, perioperative complications occurred in 18% of the operated cases (occurrence of transient diabetes insipidus with the need for desmopressin substitution in 7.1%, occurrence of an intraoperative dural tear with the need for postoperative surgical revision in 4.7%, operative revision due to postoperative hemorrhage in three cases 1.4%).

GTR was achieved in 79.1% (evaluated by analyzing the postoperative imaging and determined as no residual tumor detected on the postoperative MRI). Patients with residual (mostly suprasellar) tumor were advised to perform a follow-up MRI after 6–12 weeks. In cases with good access to the descended residual tumor, the patients underwent a second transnasal surgery.

### Cut-off values, sensitivity and specificity

**Fig. 4:**
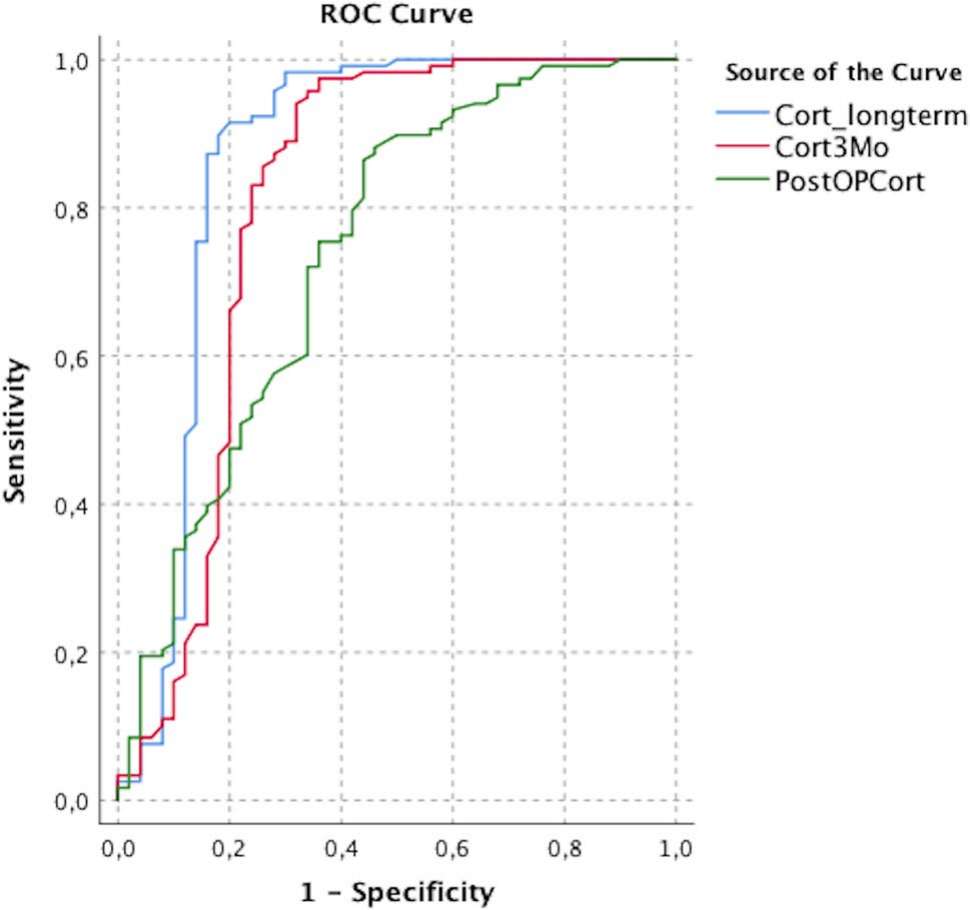
Receiver operator characteristic (ROC) curve showing the sensitivity and specificity of direct postoperative basal cortisol level, 3 months postoperatively and long-term, indicating a higher sensibility and specificity of the long-term cortisol compared to the direct postoperative cortisol level

According to the receiver operator characteristic (ROC) curves, fasting serum cortisol levels analyzed 6 weeks to 3 months after surgery were more accurate in predicting long-term intact corticotroph function (area under the curve, AUC 0.808) compared to the directly postoperative assessed levels (AUC 0.740). Naturally, the AUC of long-term basal cortisol levels predicted the corticotroph function most appropriately (AUC 0.864) (Fig. 4).

The cut-off value for direct postoperative fasting serum cortisol level was 6.9 µg/dl (sensitivity 86.4%, specificity 56%, Youden-Index 0.424). For values measured after 6 to 12 weeks after surgery, the cut-off prognostic basal cortisol was 6.95 µg/dl with a sensibility of 94.1% and specificity of 68% (Youden-Index 0.621). Basal cortisol measured after at least one year after surgery had the highest prognostic power at a value of 8.35 µg/dl (sensitivity 90%, specificity 82%, Youden-Index 0.718) (Table 2).

**Table 2:** Morning serum cortisol levels assessed on the fifth day after surgery (postoperative value), 6–12 weeks after surgery and one year after surgery

Cortisol morning serum values (ug/dl)	Normal pituitary function if cortisol greater than or equal to^a^	Sensitivity	Specificity		Youden Index
Postoperative value	2.8	**0.949**	0.32	0.269	
	6.9*	0.864	0.56	0.424	
	*10.15*	0.729	0.64	0.369	
	20.65	0.195	**0.94**	0.135	
6–12 weeks after surgery	6.85	**0.949**	0.66	0.609	
	6.95*	0.941	0.68	0.621	
	*10.05*	0.746	0.78	0.526	
	20.3	0.085	**0.94**	0.025	
1 year after surgery	7.05	**0.949**	0.72	0.669	
	8.35*	0.898	0.82	0.718	
	*10.05*	0.805	0.84	0.645	
	20.45	0.076	**0.94**	0.016	

The optimum cut-off value is marked*. The lower and higher cortisol value describes the 95% sensitivity and 95% specificity. The Youden index describes the maximum potential effectiveness of a certain cut-off value. Note that the described cut-off value determines the intact corticotropic function

Numbers in boldface determine the highest sensitivity or specificity

### Long-term corticotropic deficiency

Long-term hydrocortisone replacement after 1 year was observed in 30.9% of the patients and in 30.4% of the patients before surgical treatment. Overall, 9% recovered from preoperative hypocortisolism and 10.4% developed a new postoperative impaired HPA-axis leading to hypocortisolism. In total, 80.6% of our patients had no changes regarding the pituitary function (persistent hypocortisolism without recovery or intact function postoperatively in patients without preoperative hypocortisolism).

In 14.6%, the postoperative cortisol level was above the measured threshold of 6.9 µg/dl while patients still suffered from long-term hypocortisolism (false negative patients). False positive results occurred in 11.1% of the cases with our assessed direct postoperative cortisol cut-off value of 6.9 µg/dl (hydrocortisone substitution in patients with potentially intact HPA-axis). In total, regarding that all patients had a hydrocortisone replacement for safety reasons until the first endocrinological follow-up 6 weeks to 3 months after surgery, 66.3% of the patients underwent substitution while having a basal postoperative cortisol level above the determined threshold.

### Prognostic factors

Gender, age, year of operation, GTR, and preoperative KPS did not significantly influence the occurrence of a new postoperative hypocortisolism (*p* > 0.05). The occurrence of complications significantly increased the risk for a new postoperative corticotroph deficiency (r, 0.281; *p* < 0.01, OR 4.4, overall long-term hypocortisolism OR 2.7, *p* 0.006) and the postoperative clinical outcome in terms of KPS (KPS, r, − 0.165; *p* 0.018).

## Discussion

Compared to current literature, a new onset of hypocortisolism is described in approximately 2% up to 10% [4, 9, 11, 23, 35], depending on the surgical approach which is consistent with our presented results.

Long-term hypocortisolism requiring substitution with hydrocortisone occurred in 30.9% of all cases; in 10.4%, the deficiency was new, and 9% of the patients with preoperative hypocortisolism had intact pituitary hormone secretion at follow-up of minimum 1 year.

Our results show a higher rate of postoperative improvement of preoperative manifest hypocortisolism, as current literature reports of a laboratory normalization of 3% after 6 months for the cortisol axis. We also identified a higher rate of new hypocortisolism compared to the stated 6% of new postoperative hypocortisolism in current literature [20]. Compared to other tumors of the sellar regions, these percentages seem very low (craniopharyngiomas: up to 53% of postoperative hypocortisolism after TSS [26], and rate of hypocortisolism after resection of Rathke’s cleft cyst, 24% [25]). As these tumors were excluded from the analysis, no comparison between current literature and our results can be made concerning other sellar tumors than pituitary adenoma.

### Limitations

Our study analyzed patients retrospectively, but complete long-term follow up data was available for a considerably large series of 211 patients. Unfortunately, the burden of disease caused by long-term hormone substitution has not been evaluated yet and was not included in our data examination. Symptoms of hypocortisolism, such as fatigue and impairment of daily living activities [14], are therefore not stated or described in our study. Therefore, we cannot assess the overall burden of disease caused by the transnasal surgical resection of pituitary adenoma. We unfortunately did not evaluate the subjective disability experienced and the prospective assessment of quality of life and impairment of daily living activities would give beneficial information on the overall treatment benefit or risks for patients treated surgically for pituitary adenoma [14].

Furthermore, patients were only included and analyzed, if complete endocrinological and clinical follow-up data was available. A bias is therefore included and needs to be discussed, potentially including more patients with complications and postoperative deficiencies in the analysis because of their increased need of postoperative supervision and follow-up appointments. Therefore, the evaluated 10% rate of new postoperative hypocortisolism is most probably overrated in our patient cohort.

As we aimed to focus on hypocortisolism, we did not assess other pituitary deficiencies such as the often-frequented hypogonadism in male patients which presents a limitation of our study.

We performed a purely retrospective analysis, and patients describing symptoms of hypocortisolism after hydrocortisone withdrawal were continued on hydrocortisone substitution without further assessment, based on the assumption that ACTH testing may not detect cases of new-onset secondary adrenal insufficiency [7, 10]. The uncertainty around the clinical and the laboratory investigation of hypocortisolism remains a strong limitation of our study.

### Cut-off values and timing of analysis

While detection of intact corticotroph function is of main interest to prevent Addison’s crisis, we found different values for the sensitivity and specificity of laboratory cortisol testing direct postoperatively, 6–12 weeks after surgical treatment and 1 year after surgery. Of course, the closer the laboratory analysis coincides with the long-term follow-up clinical examination, the more sensitive and specific it becomes as some patients recover from postoperative hormone deficiencies and some patients tend to develop a secondary hypocortisolism. Our main interest is therefore to question the use of direct postoperative basal cortisol assessment if its use is very limited.

### Prediction of long-term corticotropic deficiency

The exact cut-off value of cortisol serum levels to determine hypocortisolism and its appropriate timing of testing (laboratory value measured in µg/dl in morning serum) is still matter of debate [6, 24, 38]. Published cut-off values range between 4 µg/dl [21], 8 µg/dl [6], and 14 µg/dl [21]. In our study, we classified patients based on their actual clinical need of hydrocortisone substitution regardless of their serum cortisol levels, which may over- or underestimate the real fraction of patients suffering from hypocortisolism due to false interpretation symptoms such as fatigue. The measured and assessed cortisol cut-off value of 8.35 µg/dl is congruent with and within the range of published data. Patients with basal serum cortisol levels above > 10 µg/dl were declared as “ hypocortisolism” if they still presented with symptoms of hypocortisolism and had the clinical need for hydrocortisone supplementation when the hydrocortisone administration was reduced (mostly symptoms of fatigue). False high basal cortisol levels due to either ingestion of hydrocortisone before blood testing (24 h off replacement hydrocortisone) or even factitious ingestion of hydrocortisone [1] were tried to be accounted for but cannot be excluded completely and may increase the uncertainty around a specific “cut-off” value for intact corticotroph function. Values from patients stating they had their hydrocortisone medication or food/ coffee intake before the blood was taken were of course excluded from analysis.

In our department, hydrocortisone supplementation is usually continued for 6 to 12 weeks after transsphenoidal surgery until the following endocrine laboratory testing, regardless of the assessed postoperative cortisol serum values. Side effects of supplementary (unnecessary) hydrocortisone administration could be prevented if the post-operative basal cortisol level or timing was more reliable and as early as possible, and direct postoperative testing obsolete if the positive predictive value is inferior to the long-term testing. Unfortunately, we did not assess possible consequences of supplementary (over-substituted) hydrocortisone substitution in terms of possible side effects such as high blood pressure, diabetic complications, or osteoporosis [36]. In order to assess the complete impact of false hydrocortisone administration, a prospective analysis of patients suffering from pituitary adenoma would be necessary, with a close attention to signs of under- or over-supplementation. Neither did we assess economic differences in term of cost-effectiveness of early versus late assessment of serum cortisol values and correct hydrocortisone substitution.

In total 66.3% if the patients underwent hydrocortisone substitution (substitution dosage of 10 mg administered in the morning and midday for 6 weeks) while having intact postoperative pituitary function using our defined cut-off value. The use of direct postoperative cortisol level evaluation has previously been questioned regarding its utility in clinical practice and implication for further hydrocortisone substitution [16, 19, 30, 32]. With the risk of Addison’s disease, some patient may be rather over-supplemented, with the risk of long-term effects of hypercortisolism [34]. Therefore, reliable cut-off values with a high positive predictive value and an optimal timing of analysis need to be provided. In out department, we shifted towards a more individual approach focusing on patients with very low postoperative cortisol levels and higher levels to present in our outpatient department earlier in order to adjust or stop the hydrocortisone substitution. We furthermore abandoned the strict cortisol cut-off value of 10ug/dl and focus more on the clinical and functional status of the patient after with-drawing the hydrocortisone substitution. Nevertheless, the higher mortality caused by hypocortisolism [12] compared to risks caused by over-supplementation of hydrocortisone may shift the optimal early cut-off value for basal cortisol towards a higher sensibility at the cost of a lower specificity.

Hypocortisolism may be caused by surgery related injury of the pituitary gland, but also due to long-term administration of corticoids. In our cohort population, patients obtained only 20 mg of oral hydrocortisone per day, which is usually below the threshold causing HPA suppression [22]. Only one patient was given prednisolone for rheumatoid arthritis prior to pituitary surgery and was therefore excluded from the analysis.

## Conclusion

In 30.9% of all patients, long-term hydrocortisone substitution was necessary to support the adrenocorticotropic axis in patients with hypocortisolism. Prediction of hormone production, especially hypocortisolism, remains an important matter of debate in the neurooncological context; the duration of hydrocortisone substitution and timing of evaluation should therefore be questioned. In our study, evaluated cut-off values were congruent with published literature, and substitution in patients with intact postoperative pituitary function should be discussed further to reduce the risk of potential side effects. An improvement in reliability and prognostic quality of laboratory testing may reduce hydrocortisone oversupply without increasing the amount of undetected postoperative hypocortisolism.

## Data Availability

The datasets used and/or analyzed during the current study are available from the corresponding author on reasonable request.
